# Myokines in cancer: bridging molecular mechanisms to biomarker discovery and therapeutic innovation

**DOI:** 10.3389/fonc.2026.1751316

**Published:** 2026-04-21

**Authors:** Huizhe Zhang, Dongjing Fu, Meijing Yue, Fen Cheng, Ying Jiang, Juan Zhao, Wenjuan Wang, Shuqing Deng, Dan Tian, Gaowa Jin, Quanfu Li

**Affiliations:** 1 Department of Medical Oncology, Ordos Central Hospital, Ordos, China; 2 Department of Pathology, Harbin Medical University, Harbin, China; 3 Ordos School of Clinical Medical Inner Mongolia Medical University, Inner Mongolia, China; 4 Baotou Medical College, Inner Mongolia University of Science and Technology, Baotou, China

**Keywords:** biomarkers, cancer, myokines, pathway mechanism, therapeutic targets

## Abstract

Exercise can regulate the physiological functions of the body by inducing the secretion of myokines, which are bioactive factors mainly secreted by muscle cells. This review classifies myokines based on their functional characteristics, including metabolic regulation (such as myostatin, interleukin-6), neuroregulation (brain-derived neurotrophic factor), cell proliferation/differentiation regulation (myogenic proteins), immune regulation (tumor necrosis factor- alpha), and factors involved in angiogenesis and extracellular matrix remodeling (such as connective tissue growth factor).Cancer, as a consuming disease, often accompanies muscle atrophy and depletion in its advanced stage, thereby affecting the normal secretion of myokines. Increasing research evidence indicates that myokines play a dual regulatory role in the occurrence and development of cancer. Some myokines (such as interleukin-6, tumor necrosis factor- alpha) have environment-dependent functions and can exhibit pro-cancer or anti-cancer effects depending on the microenvironment; while factors such as myostatin show stable anti-tumor potential by regulating key molecular pathways such as the PI3K/AKT pathway, epithelial-mesenchymal transition, and HIF-1α. It is worth noting that muscle cell factors can indirectly influence the disease outcome of cancer by regulating key cells and structures in the tumor microenvironment (such as tumor-associated macrophages, regulatory T cells, and cancer-associated fibroblasts), as well as by participating in the angiogenesis process. At the clinical application level, muscle cell factors are expected to become potential biomarkers for cancer diagnosis and prognosis assessment (such as elevated irisin levels in patients with renal cancer and elevated interleukin-6 levels in patients with bile duct cancer). They also have great potential as therapeutic targets. For example, MSTN inhibitors can effectively alleviate cancer cachexia symptoms, and the combination of anti-interleukin-6 treatment with immune checkpoint blockade therapy can produce a significant synergistic therapeutic effect. This review systematically summarizes the latest research progress on the molecular interaction mechanisms mediated by myokines in cancer, emphasizing their potential for translational applications in precision oncology. Myokines not only regulate the physiological functions of the musculoskeletal system, but also have a close association with the occurrence and development of cancer. The intrinsic connection between myokines and muscle atrophy as well as cancer-related cachexia still requires further in-depth exploration. As emerging biomarkers, myokines can be combined with various diagnostic and therapeutic techniques, which is expected to further improve the survival rate of cancer patients, protect muscle function, and also provide new research ideas for exploring the interrelationship between muscles and cancer and the pathogenesis of related muscle diseases.

## 1 Introduction

### 1.1 Factors of muscle and muscle physiology and the initiation, progression and treatment of cancer

#### 1.1.1 Interaction between muscle and tumor: bidirectional channel

The interaction between skeletal muscle and tumor is not one-way; instead, it represents a complex bidirectional interaction that determines the clinical development trajectory of cancer patients. On one hand, tumors and their related microenvironments release pro-inflammatory cytokines and catabolic factors (such as tumor necrosis factor- alpha), which act on skeletal muscle, triggering the ubiquitin-proteasome system and autophagy-lysosome pathways, thereby causing muscle atrophy - this condition is called cancer cachexia. This “tumor-to-muscle” signal transmission is an important factor contributing to the severity of the disease, it reduces the tolerance to chemotherapy and increases the mortality rate.

On the other hand, the “muscle-to-tumor” signaling axis is mediated by myokines, which can directly affect the dynamics of tumor cells or indirectly reshape the tumor microenvironment (TME). There is evidence that exercise-induced myokines can enhance immune surveillance, inhibit angiogenesis, and induce malignant cell apoptosis. For example, serum collected from individuals after exercise has been proven to inhibit the proliferation of breast cancer and prostate cancer cell lines *in vitro*, and this effect is attributed to the acute increase in specific myokines. However, this relationship is complex; certain myokines exhibit different action patterns under different physiological environments, the malignancy of tumors, and the specific receptor characteristics of tumor cells.

#### 1.1.2 Muscle and myokines

Myokines are a class of bioactive peptides or proteins secreted by muscle tissues (including skeletal muscle, cardiac muscle, etc.), and muscles can secrete more than 650 myokines, exerting regulatory effects through paracrine, autocrine, and other three ways, constructing a communication network between muscles and various organs throughout the body. Myokines can precisely regulate a variety of key physiological functions such as energy metabolism, inflammatory response, cell proliferation and differentiation, maintenance of muscle homeostasis, and regulation of immune balance, and play a crucial role in maintaining the stability of the body’s physiological internal environment, ensuring the normal physiological functions of muscles and responding to various physiological and pathological states.

#### 1.1.3 Myokines and cancer

Skeletal muscle is the physical barrier of the body. Healthy muscle tissue has the characteristics of low matrix hardness, high oxygen, and dense extracellular matrix (ECM), which can inhibit the colonization and invasion of tumor cells; while in the state of sarcopenia: muscle ECM remodeling: collagen and fibronectin expression decreases, the “density” of muscle tissue decreases, tumor cells are more likely to penetrate the muscle barrier and metastasize to distant organs (such as lungs, liver, bones); muscle angiogenesis increases: the repair process after muscle injury will induce local angiogenesis, tumor cells can enter the blood circulation through these new blood vessels and accelerate distant metastasis. Cancer patients often become emaciated during the progression of the disease, on the one hand, cancer consumes the body’s muscles and nutrients to ensure the unlimited proliferation of cancer cells; on the other hand, the side effects of cancer treatment drive muscle loss and muscle consumption. And skeletal muscle is the main source of myokines, which then leads to a decrease in myokine secretion and a decline in the regulatory ability of cancer. At the same time, muscle loss increases the risk of precancerous lesions and worsens prognosis.

**Figure 1:**
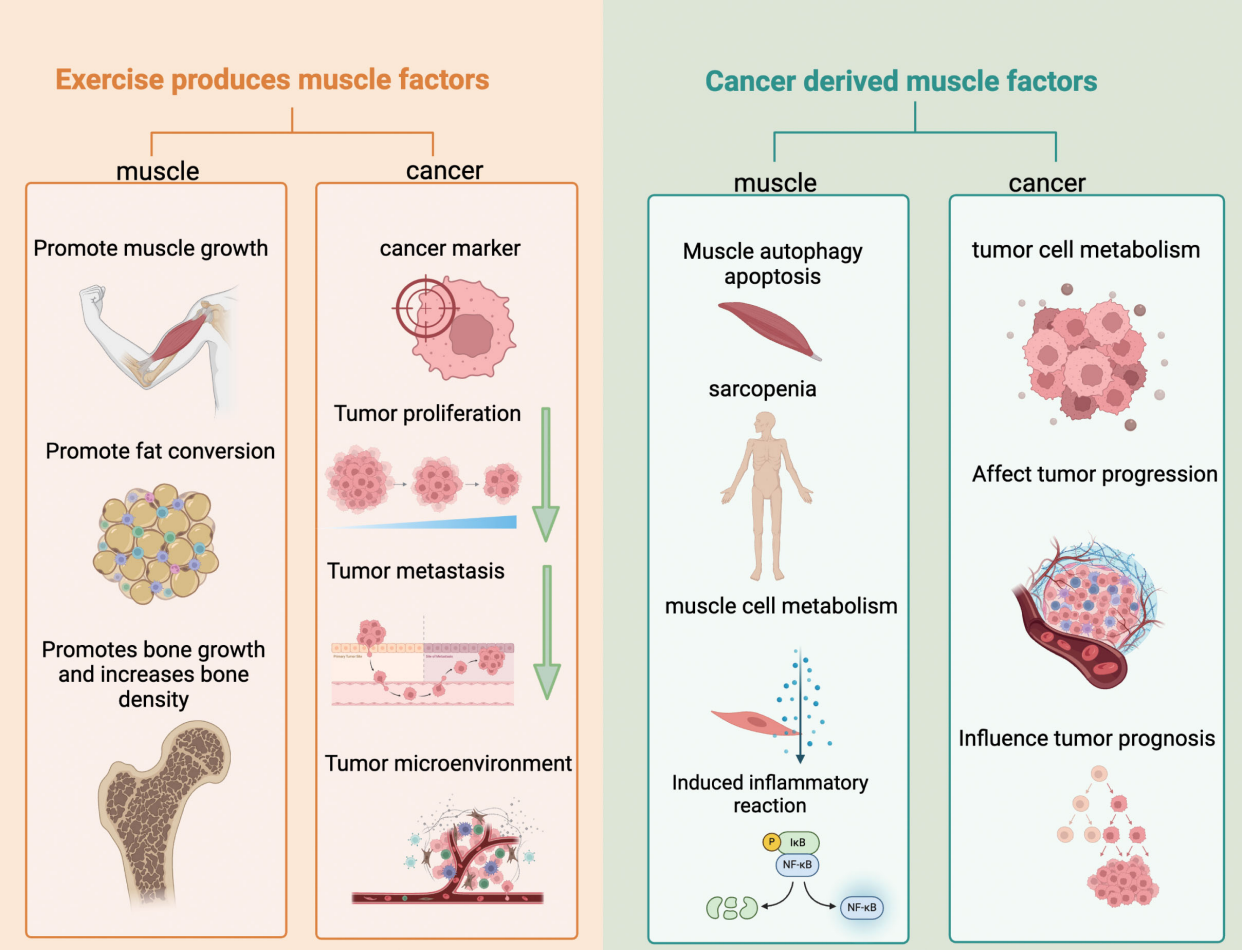
Bidirectional regulation of myogenic factors in cancer the role of myogenic factors in cancer is driven by two sources, forming a bidirectional regulatory network: Myogenic factors induced by exercise: On the one hand, they promote muscle growth, fat transformation, and bone density improvement, maintaining muscle and bone health; on the other hand, they can reduce the expression of cancer markers, inhibit tumor proliferation and metastasis, and improve the tumor microenvironment, exerting an anti-cancer effect. Myogenic factors derived from cancer: On the one hand, they induce muscle autophagy, apoptosis, and inflammatory responses, leading to muscle dysfunction such as sarcopenia, and affecting muscle cell metabolism; on the other hand, they regulate the metabolism of tumor cells, promote tumor progression, and affect the prognosis of patients, demonstrating the dual role of myogenic factors in the interaction between cancer and muscle.

Studies have proved that myokines can regulate various biological behaviors of malignant tumor cells, such as fibroblast growth factor 21 (Fibroblast Growth Factor 21, FGF21), which inhibits the invasion and metastasis of pancreatic ductal adenocarcinoma by inhibiting interleukin-17A, and FGF21 is also a new “secreted immune checkpoint”, which regulates the cholesterol metabolism of CD8-positive T cells, causing the exhaustion of CD8-positive T cell function, thereby limiting the anti-tumor immune response (4). Tumor necrosis factor-alpha (TNF-α) itself has a direct killing effect on tumor cells and can induce apoptosis and necrosis of tumor cells. At the same time, it can also enhance the recognition specificity and killing activity of immune cells against tumor cells by activating the immune system, thereby exerting biological figure 1 effects such as inhibiting tumor growth and blocking tumor metastasis (**Figure 1**).

##### 1.1.3.1 Myokines and cancer diagnosis

Myokines, as a class of protein/polypeptide signaling molecules secreted by skeletal muscle, have specific expression patterns that change with tumor occurrence, progression, and metastasis, and can be stably present in body fluids such as peripheral blood and saliva, becoming new non-invasive biomarkers for early cancer screening, type differentiation diagnosis, and prognosis stratification; at the same time, some myokines can be combined with traditional tumor markers to significantly improve the sensitivity and specificity of diagnosis. Combining the clinical needs of cancer diagnosis for early detection, precise classification, and prognosis prediction. Cancer early screening can incorporate skeletal muscle index (SMI) into the risk assessment of precancerous conditions, and individuals with sarcopenia need to strengthen cancer screening and lifestyle intervention. Myokines assist in cancer diagnosis.

##### 1.1.3.2 Myokines and cancer progression

Myokines are proteins secreted by skeletal muscle, and most of them exert anti-cancer effects through anti-inflammatory, immune activation, inhibition of proliferation and angiogenesis, etc., while exercise can significantly enhance their anti-cancer effects; however, studies have shown that myokines exhibit bidirectional regulatory characteristics in tumor progression, which can either exert anti-cancer effects or show pro-cancer effects. Myokines can participate in the regulatory process of tumor progression through bidirectional regulation with the tumor microenvironment. The myokines induced by exercise can exert anti-tumor effects by inhibiting tumor proliferation, migration, EMT, and angiogenesis; at the same time, the interaction between myokines and tumor-derived factors can induce muscle wasting cachexia and accelerate tumor progression. Cachexia is a common complication of cancer, mainly manifested as a decrease in skeletal muscle mass, and traditional nutritional therapy cannot reverse it [40]. Cachexia can cause muscle atrophy, seriously affecting the prognosis and survival rate of cancer patients. It is estimated that about 30% of cancer patients eventually die from cachexia. Muscle atrophy is a serious clinical problem involving the loss of muscle mass and strength, often developing with pancreatic cancer, lung cancer, and gastric cancer, etc. Studies have confirmed that myokines IL-6 and TNF-α also play important roles in the mechanism of cachexia. In particular, TNF-α can directly have a catabolic effect on skeletal muscle and can induce muscle atrophy by inducing ubiquitin-protease activation [42]. MSTN can promote the process of muscle wasting and cachexia, and is one of the causes of shortened survival time and decreased quality of life in cancer patients. Studying the mechanism of myokines regulating cancer cachexia can not only find new targets for intervention in the progression of cancer patients’ conditions, but also provide new ideas for the prevention and treatment of muscle wasting and cachexia. At the same time, combined with immunotherapy treatment, it can significantly enhance the efficacy of immunotherapy, such as the combination therapy of IL-6 and PD-L1 antibody can effectively inhibit tumor progression in mouse models of pancreatic cancer [43].

##### 1.1.3.3 Myokines and cancer treatment

Cancer treatment methods mostly adopt surgery, radiotherapy and chemotherapy drugs (paclitaxel). During treatment, patients may experience muscle atrophy and weight loss, and in severe cases, they may develop cachexia. Myokines are secreted by muscle tissue, and the myokines produced by exercise can exert regulatory effects on tumors. Muscle atrophy will affect the production of myokines. Therefore, cancer treatment combined with improving muscle atrophy and strengthening exercise can amplify the therapeutic effect. Exercise intervention is the only non-pharmacological method that can reverse cancer-induced sarcopenia. It mainly consists of resistance training combined with aerobic training, which is the core approach for protecting the muscle mass and function of cancer patients, and can directly inhibit tumor progression. Resistance training (such as dumbbells, elastic bands): By activating the Akt-mTOR pathway, it promotes muscle protein synthesis, inhibits MAFbx/MuRF1 expression, and reverses muscle atrophy; at the same time, it can promote the secretion of irisin and IL-6 (anti-inflammatory type), directly inhibiting the proliferation and metastasis of tumor cells; aerobic training (such as brisk walking, jogging): Improves overall metabolism, reduces systemic inflammation induced by tumors, and enhances the sensitivity of tumor cells to chemotherapy; there is clinical data evidence: Cancer patients who undergo 12 weeks of resistance training can increase SMI by 5%-10%, increase the chemotherapy completion rate by 40%, and prolong the progression-free survival (PFS) by more than 20%.

At the same time, nutritional support is provided: precise supplementation to maintain the competitive advantage of muscle synthesis and nutrition, supplementing muscle synthesis raw materials, providing a foundation for muscle synthesis. Supplementing high-quality protein efficiently activates the muscle mTOR pathway, promoting protein synthesis; further supplementing targeted amino acids with glutamine to alleviate muscle glutamine loss. Tumors induce muscle protein degradation, inhibition of synthesis, and ultimately trigger cancer-related sarcopenia (specifically reduced muscle mass and decreased function due to disease) and even progress to cancer cachexia (sarcopenia + weight loss ≥ 5% + anorexia/inflammation), which is the core manifestation of muscle damage during cancer progression and occurs earlier than the visible clinical weight loss. Combining intervention treatments of exercise + nutrition + anti-tumor treatment to achieve “1 + 1 + 1 > 3” basic intervention and anti-tumor treatment combination is the core direction for the future: For example, chemotherapy combined with resistance training + whey protein supplementation can significantly reduce chemotherapy-related muscle damage, increase the chemotherapy completion rate, and simultaneously enhance the killing effect of chemotherapy on tumor cells; immune checkpoint inhibitors combined with irisin supplementation can restore anti-tumor immunity and improve the response rate of ICIs.

### 1.2 Types of muscle factors and their physiological functions

#### 1.2.1 Metabolic regulation related muscle factors—irisin

Exercise has the power to alter human metabolism, and one of the elements influencing metabolism is the muscle factor produced during exercise (1). Irisin is a muscle factor that primarily plays a metabolic role, stimulating adipose cell browning, aiding in the treatment of obesity and related metabolic illnesses, and regulating hepatic glucose metabolism (2). Meanwhile, irisin is classified as either glycosylated or non-glycosylated. Post-translational modifications to irisin appear to modify its function (3). However, glycosylation had no effect on irisin’s anticancer activity. Irisin is closely related to cancer, exhibiting different expression levels in a variety of cancers, and is involved in cancer occurrence, development, metastasis, and treatment via a variety of mechanisms, making it a promising biomarker and potential target for cancer diagnosis and therapy (4). Irisin is expressed differently in different malignancies. Some studies have discovered that Irisin expression is elevated in gastrointestinal cancer, ovarian cancer, cervical cancer, thyroid cancer, pancreatic cancer, kidney cancer, and other cancers. For example, the level of Irisin found by immunohistochemistry in gastrointestinal cancer tissues is substantially higher than that of normal tissues. Certain pathologic kinds of thyroid cancer (eosinophilic papillary carcinoma, undifferentiated carcinoma) are distinguished from normal thyroid tissue and other types of thyroid cancer (5). However, in certain investigations on breast cancer, colorectal cancer, liver cancer, and other cancers, serum Irisin levels were found to be lowering as compared to healthy persons, with serum Irisin levels in breast cancer patients being considerably lower than healthy controls (6, 7).

#### 1.2.2 Metabolic regulation related muscle factors—IL-6

IL-6 produced by muscle plays a significant role in exercise stress, metabolic control, immune regulation, and maintenance of muscle physiological function. Involved in the repair and regeneration of muscle following damage. In order to preserve the structural and functional integrity of muscles, IL-6 plays a crucial regulatory role in the repair of microinjuries brought on by exercise (8, 9). During exercise, the muscles release interleukin-6, which stimulates the liver to do gluconeogenesis and glycogenolysis, raising blood sugar levels. This technique is one of the ways that muscles can constantly receive energy sources. In the state of insulin resistance, IL-6 secreted by muscle may participate in the abnormal regulation of blood glucose homeostasis, but the specific mechanism is complicated (10), which may be related to the interference of IL-6 on insulin signaling pathways and the influence on adipose tissue metabolism. By triggering pertinent signaling pathways in hepatocytes, including phosphorylated glycogen synthetase kinase-3 (GSK-3), it prevents glycogen synthesis and encourages glycogen breakdown into glucose. Muscle-secreted IL-6 may also contribute to the aberrant regulation of blood glucose homeostasis in the state of insulin resistance. Like Irisin, IL-6 regulates lipid metabolism and encourages fat intake. Free fatty acid (FFA) release is also increased by IL-6(Lima 11).By stimulating hormone-sensitive lipase (HSL) in fat cells, it converts triglycerides into FFA and glycerol, raising blood levels of FFA and giving muscles and other tissues a source of energy (12).Furthermore, IL-6 might also have an impact on the metabolic remodeling of adipose tissue via controlling adipocyte development and function. The imbalance of IL-6 released by muscle and adipose tissue may exacerbate the problem of lipid metabolism in obesity and related metabolic disorders. For participate in the body’s immune response and control the severity and length of the inflammatory response, IL-6 can also be utilized as an immunomodulator (13).

#### 1.2.3 Neuromodulation related muscle factors—BDNF

A significant member of the neurotrophic factor family, BDNF is primarily produced and secreted by neurons (14), but new research has shown that muscle cells can also release BDNF in response to stimuli like exercise. A fundamental protein that is essential for neuron survival, development, differentiation, and functional maintenance is encoded by the gene, which is found on human chromosome 11. BDNF has the ability to control glucose metabolism and can also play a role in controlling the body’s overall glucose metabolism process (15). By increasing insulin sensitivity and encouraging glucose uptake and utilization, it may help muscle maintain glucose metabolic homeostasis. By controlling the neuroendocrine system and inflammatory response10, BDNF may also indirectly affect the metabolism of glucose in the liver and adipose tissue and preserve blood glucose balance. BDNF can control the expression of genes linked to fat metabolism in adipose tissue, such as leptin, encourage fatty acid oxidation and fat breakdown, and lessen fat formation (16). In neuroprotection and neuroplasticity, BDNF serves to support neuron development and survival (17). Muscle-secreted BDNF can influence peripheral nerves, support neuron growth and survival, and control neuroplasticity. One of the main elements controlling neuroplasticity is BDNF (18). It plays a role in long-term inhibition (LTD) and long-term enhancement (LTP) in the central nervous system. One kind of growth factor that is essential to the proper development of the brain is brain-derived neurotrophic factor (BDNF). In addition to its important function in the neurological system, BDNF also plays a part in muscle growth and repair, helps create and maintain neuromuscular junctions, enhances muscle contraction and endurance (19), and may even have a role in the development of tumors, according to some findings (20).

#### 1.2.4 Muscle factors associated with cell proliferation and differentiation—MSTN

MSTN is a member of the transforming growth factor-β (TGF-β) class (also known as GDF8), is primarily released by skeletal muscle cells (21). The gene is located on human chromosome 2 and codes for a secreted glycoprotein. The amount of MSTN expressed in muscle is influenced by a great many factors, including age, hormone levels, food, and physical exercise, MSTN is activated by changing its structure. Exercise, especially strength training, can reduce MSTN expression, but extended periods of inactivity or muscle disuse may increase it. By inhibiting muscle cell proliferation and differentiation, MSTN, a major negative regulator of muscle growth, can limit muscular growth and development (Ozcan 22, 23). Muscle atrophy and weakening will result from an increase in its expression level in aging and muscle disorders. Inhibition of GDF8 resulted in significant muscle growth in mice, but less so in humans. Extensive TGF-β inhibition can promote muscle growth, of which gdf8 specific inhibition is critical (24). At the same time, myostatin is strongly associated with cachexia and cancer muscle atrophy. Myostatin expression and biological activity were found to be up-regulated in experimental cancer cachexia in related research (25). In two ways, myostatin can exacerbate tumor circumstances and result in a bad prognosis: on the one hand, myostatin secretion accelerates cachexia and encourages TAM migration and the cancer process; on the other hand, myostatin causes muscle atrophy (26), which in turn promotes the development of cancer and sarcopenia (27).

#### 1.2.5 Muscle factors associated with immune regulation—TNF-α

Although muscle cells can secrete TNF-α, it is generally thought of as an inflammatory factor (28). While an adequate amount of TNF-α has a regulatory effect on muscle cell survival and differentiation, excessive secretion will increase muscle catabolism, resulting in muscle atrophy and other issues, and TNF-α can affect fat metabolism and transformation (29). Notably,TNF-α promotes skeletal muscle atrophy and specifically inhibits myoblast differentiation (30). Through a variety of signaling pathways, tumor necrosis factor-α (TNF-α) affects the proliferation, apoptosis, migration, invasion (31), and other processes of tumor cells, as well as interacting with cells in the tumor microenvironment. It also has the dual effects of either promoting or inhibiting tumor in different types of cancer (32), affecting the proliferation, apoptosis, migration, invasion and other processes of tumor cells through a variety of signaling pathways (33), and interacting with cells in the tumor microenvironment. It has a major impact on the onset, progression, and management of cancer. Numerous factors, including exercise, oxidative stress, inflammatory activation, and muscular injury, influence the release of TNF-α in muscles. The mechanism behind the elevation of TNF-α expression in muscle caused by prolonged or high-intensity exercise may involve the buildup of muscle metabolites, modifications in calcium ion concentration, alterations in mitochondrial function, and the activation of exercise-induced neuroendocrine signals. TNF-α, which is released by activated immune cells, can influence neighboring immune cells in terms of immunological control (34). Their immunological function is activated by T cells, B cells, macrophages, and other cells. It can enhance the cytotoxic effect of T lymphocytes (35), encourage their activation, proliferation, and differentiation, and improve their ability to identify and eliminate tumor cells or pathogens; Encourage B cells to participate in the humoral immune response and generate antibodies; TNF-α can also activate macrophages, improve their phagocytic and antigen-presenting capabilities, encourage the onset and progression of an inflammatory response, and contribute significantly to the body’s immune defense mechanism (36). TNF-α is one of the important nodes in the immune cytokine network that controls the intensity and duration of the immune response by either inducing or inhibiting the production of other cytokines TNF-α is a key inflammatory initiator in the local muscle tissue (37). It can cause inflammation, cause immune cells and plasma proteins to seep into the tissue space, and enhance the permeability of vascular endothelial cells. TNF-α can also cause inflammatory cells to migrate to the site of damage or infection, such as neutrophils and monocytes, which exacerbates the inflammatory response. In conditions like viral myositis and muscle strain, muscles release more TNF-α, which can contribute to muscle atrophy and cause symptoms including tissue edema, discomfort, and local inflammatory cell infiltration.

#### 1.2.6 The extracellular matrix regulates related muscle factors—CTGF

The CCN family includes a cysteine-rich secreted protein, known as connective tissue growth factor (CTGF) (38). The gene, which codes for a protein with a molecular weight of roughly 36–38 kDa, is found on human chromosome 6. It has the ability to encourage the production and accumulation of. Numerous factors control the secretion of CTGF in muscle tissue, which is crucial for preserving the mechanical stability and integrity of muscle tissue. On the other hand, malfunction and muscle fibrosis could result from aberrant expression. The secretion of muscle CTGF may also be regulated by changes in hormone levels and inflammatory cytokines. And enhance the extracellular matrix’s deposition in muscle cells and muscle satellite cells. CTGF, activated in muscular tissue, has been linked to the initiation and spread of malignancies. There is mounting proof that CTGF controls cancer cell motility, invasion, angiogenesis, and death. May encourage the production of extracellular matrix: One of the main modulators of the metabolism of the extracellular matrix (ECM) is CTGF. It can enhance extracellular matrix deposition and encourage the production of collagen, fibronectin, and other extracellular matrix constituents in fibroblasts, smooth muscle cells, and muscle satellite cells. CTGF stimulates associated signaling pathways in muscle tissue, including the phosphatidylinositol 3-kinase (PI3K-AKT) and mitogen-activated protein kinase (MAPK) pathways. CTGF participates in the extracellular matrix remodeling process in addition to encouraging the formation of extracellular matrix. It influences the breakdown and renewal of extracellular matrix components and controls the expression and activity of matrix metalloproteinases (MMPs) and their inhibitors (TIMPs). CTGF can facilitate the repair and regeneration of injured tissues and balance the synthesis and breakdown of extracellular matrix in the context of muscle injury or disease. For instance, CTGF expression is elevated during the healing process following muscle trauma. CTGF can stimulate angiogenesis, which is crucial for the vascular growth and repair of muscle tissue, by controlling the activities of MMPs and TIMPs. By interacting with angiogenic factors like vascular endothelial growth factor (VEGF), it can promote endothelial cell migration, proliferation, and lumen creation.

### 1.3 Mechanism of action of muscle factors on cancer cells

#### 1.3.1 Mechanism of action of Irisin on cancer cells

Irisin is a proteolytic cleavage product of the transmembrane protein fibronectin III domain containing 5 (FNDC5). Its secretion is stimulated by PGC-1α, which is the main regulator of mitochondrial generation and is activated with muscle contraction. Physiological studies have established that irisin is a driving factor for the “browning” of white adipose tissue - upregulating the dissociation protein 1 (UCP1) to promote heat production and energy consumption. In the context of cancer, this metabolic regulation holds profound significance (39). Cancer cells typically undergo the Warburg effect, shifting metabolism from oxidative phosphorylation to aerobic glycolysis to support rapid proliferation. Irisin seems to counteract this effect by reprogramming cellular metabolism back to the oxidative state, thereby depriving tumors of the need for glycolytic intermediates for biomass accumulation. Regulation of the PI3K/AKT/mTOR axis: A tumor suppressor factor dependent on the environment, the PI3K/AKT/mTOR pathway is a key regulator of cell survival and proliferation, often dysregulated in cancer. Irisin has been shown to effectively inhibit this pathway in various malignant tumors, which contrasts sharply with the pro-survival effects of other growth factors. Pancreatic cancer and breast cancer: In pancreatic cancer cell lines (PANC-1, BxPC-3), dose-dependent treatment with irisin can reduce the phosphorylation level of AKT (p-AKT), while not altering the total AKT level (40). This inhibitory effect suppresses downstream mTOR kinase (41), leading to cell cycle arrest in the G0/G1 phase. Additionally, this blockade alters the balance of Bcl-2 family proteins, downregulating the anti-apoptotic protein Bcl-2 and upregulating the pro-apoptotic protein Bax, making cells more sensitive to apoptosis and chemotherapeutic drugs such as doxorubicin. Epithelial-mesenchymal transition (EMT): Irisin also targets the metastasis cascade by inhibiting the PI3K/AKT/Snail pathway (42, 43). In lung cancer and osteosarcoma, irisin treatment inhibits Snail, a transcription factor that represses the expression of E-cadherin. By restoring the level of E-cadherin, irisin can maintain the epithelial cell phenotype and prevent its transformation into a migratory mesenchymal state, thereby reducing invasiveness. Exception in hepatocellular carcinoma (HCC): Although irisin has an inhibitory effect in most solid tumors, it has been reported to promote the proliferation and invasion of hepatocellular carcinoma by activating the PI3K/AKT pathway. This difference highlights the special importance of the tissue environment - specifically receptor characteristics (possibly integrin αVβ5) and the unique metabolic requirements of the liver, which may utilize irisin-mediated signaling to maintain rather than inhibit (44, 45).

#### 1.3.2 Mechanism of action of IL-6 on cancer cells

The effects of IL-6 from different sources on cancer vary. We focus on the role of the muscle-derived cytokine IL-6 in influencing cancer. In lung cancer, studies have shown that inflammation and the immunosuppressive tumor microenvironment are correlated with low muscle mass, further linking muscle, inflammation, and the tumor microenvironment (Alizadeh 46, 47) IL-6 prevents cancer by improving insulin sensitivity, stimulating the production of anti-inflammatory cytokines, mobilizing immune cells, and reducing DNA damage in early malignant tumor cells. IL-6 is released from contracting skeletal muscles during exercise to regulate short-term energy supply and is rapidly cleared from the plasma after exercise stops (48). The muscle-derived IL-6 enhances insulin sensitivity, stimulates the production of anti-inflammatory cytokines, reduces the proliferation and DNA damage of cancer cells, and stimulates the infiltration of tumor cytotoxic immune cells in mice. Based on these findings, we believe that IL-6 plays a key role in the various health benefits of exercise, including the prevention of cancer.

The muscle-derived IL-6 can exert anti-inflammatory effects and can also regulate inflammation to further regulate cancer In contrast, the IL-6 continuously produced by inflammatory sites by white blood cells promotes tumor occurrence by promoting chronic inflammation and activating tumor-promoting signaling pathways. Studies have shown that one of the sources of increased IL-6 in the tumor microenvironment is tumor-associated fibroblasts, and IL-6 regulates immune cells CD8 + T cells within tumor cells, thereby promoting tumor growth and immunosuppression. Through co-culture of cancer cells with fibroblasts, the trend of increased IL-6 secretion was observed (49). IL-6 regulates various tumors and has a promoting or inhibitory effect on cancer [51]. In the cancer environment, the self-expression level of IL-6 is high, and the further increase in the level of IL-6 in the circulation has become a central factor promoting tumor growth and metastasis. In contrast, exercise muscles secrete IL-6 cytokines, which inhibit tumor growth. In colon cancer, the expression level of IL-6 in patients’ bodies increases systemically, indicating a poor prognosis (50). IL-6 can regulate the state of immune cells in the tumor microenvironment and promote the metastasis and colonization of colorectal cancer cells. Animal experiments have also confirmed this, where the co-culture of colon cancer and breast cancer cells with IL-6 overexpressing mice models showed changes in immune cell states, thereby promoting the metastasis of tumor cells in the liver and lungs. IL-6 can regulate immune cells and, in the tumor immune microenvironment, regulates the process of the tumor through CD8 + T cells. In cholangiocarcinoma, IL-6 reduces the number of CD8 + T cells in the tumor microenvironment to decrease the secretion of Th1 cytokines, promote the expression of PD-1, inhibit the immune response of CD4 + T cells, regulate their differentiation, and induce their differentiation into Th2 subtypes. At the same time, it can promote the accumulation and activation of regulatory T cells and myeloid suppressor cells, inhibit anti-tumor immune responses, and facilitate tumor immune escape. Further combining IL-6 antibodies with immunotherapy, such as the combination of IL-6 antibodies and PD-L1 antibodies, inhibited tumor growth, still through regulating the activation and infiltration of CD8 + T cells to regulate the tumor (51, 52).

When IL-6 is overexpressed, blocking the IL-6 signaling pathway may have therapeutic effects on cancer (53). By inhibiting the expression of IL-6 in cancer through drugs to regulate the progression of cancer, in endometrial cancer, using Stat3 inhibitors can suppress the increase in IL-6 expression levels in the cancer, and further inhibit the migration and invasion of endometrial cancer cells. In multiple myeloma, prostate cancer, ovarian cancer, lung cancer and other cancers, IL-6 antibodies have also been proven to inhibit tumor development to a certain extent. In addition, some drugs, such as steroids, non-steroidal anti-inflammatory drugs, estrogens, cytokines, can regulate the production of IL-6, while new targeted therapeutic drugs, such as toxins binding to IL-6, monoclonal antibodies targeting IL-6 or IL-6R, and small molecule inhibitors, are still in the research stage (54).

The expression of IL-6 is related to the drug resistance of cancer 61. In bladder cancer, IL-6 is the main activator of STAT3. IL-6 promotes the proliferation, migration and invasion of cancer cells and the epithelial-mesenchymal transition (EMT) process by activating the JAK-STAT3 signaling pathway. In addition, IL-6 can also activate the RAS-MAPK and PI3K-AKT pathways, regulating the formation and development of tumors by inhibiting cell apoptosis.

#### 1.3.3 Mechanism of action of BDNF on cancer cells

BDNF affects a variety of cancers, promotes tumor cell proliferation, migration, invasion and survival, and inhibits cell apoptosis. Starting from the BDNF receptor, BDNF can bind to multiple receptors, can bind to p75 neurotrophic factor receptor (NTR) and integrin α9β1 with low affinity, and can bind to BDNF and TrkB leading to the dimerization of the receptor. In related expression profiles, it has been recognized that a variety of micrornas (miR) play a role in regulating BDNF/TrkB pathway. By binding to high-affinity receptor TrkB (55), they activate downstream signaling pathways such as PI3K/AKT, RAS/ERK, PLC/PKC, AMPK/ACC and JAK/STAT (56). Promote tumor cell proliferation, migration, invasion and survival, inhibit cell apoptosis. Provides a potential biological mechanism by which targeted therapies may be associated with reduced BDNF expression in cancer. Studying drugs that target BDNF receptors and related signaling pathways and interfere with related carcinogenic effects, abnormal BDNF signaling has been associated with the development of multiple tumors (57). (BDNF) The effects of BDNF on cancer mainly involve various pathways PI3K/Akt, Ras-Raf, PAR2, STAT3, BDNF/TrkB, such as brain-derived neurotrophic factor (BDNF) -TrKB signal. Modulating tumor-endothelial cell interactions and influencing the outcome of triple-negative breast cancer (55). In colon cancer cells, cell migration can be promoted by modulating VEGF/HO-1 activation related signaling pathways (58). The BDNF/TrkB signaling pathway becomes a potential target for cancer therapy. For example, TrkB inhibitors can inhibit the proliferation, migration and invasion of tumor cells, which has potential application value in the treatment of lung cancer, breast cancer and other cancers. New targeted therapeutic drugs, such as antibodies against BDNF or its receptors and small molecule inhibitors, are also in the research stage and are expected to provide new strategies for cancer treatment. BDNF is also associated with other diseases and factors in cancer, and it has been found that tumor necrosis factor receptor 2 (TNFR2) may play an important role in mediating the comorbidities between lung cancer tumor growth and schizophrenia-like behavior by regulating TrkB dependent BDNF levels66. In TNFR2 knockout mice, lung cancer tumor growth and schizophrenic behavior were significantly reduced, while BDNF and TrkB expression levels were reduced in tumor tissue and the prefrontal cortex of the brain. Exogenous injection of BDNF can increase tumor growth and schizophrenic behavior, and increase the expression levels of BDNF and TrkB, suggesting that TNFR2 may be a potential target linking cancer and schizophrenia, in which BDNF plays a key mediating role. It provides a new perspective for further understanding the mechanism of the association between cancer and mental diseases.

#### 1.3.4 Mechanism of action of MSTN on cancer cells

gastric cancer exhibit relatively specific down-regulation of MSTN mRNA expression in their skeletal muscle, and this early alteration is instructive for developing prevention and therapy methods for cancer cachexia (59). Inhibiting MSTN has been shown to raise reactive oxygen species and cause cancer cells to undergo apoptosis in human cervical carcinoma, offering a novel approach to the disease’s treatment (60).By preventing protein synthesis and promoting protein degradation is yet unknown how myostatin’s recruitment of TAM into the tumor microenvironment and its promotion of cancer progression relate to the biochemical mechanism by which myostatin expression in lung cancer tissues causes muscle loss and accelerates tumor growth. Myostatin signaling is elevated in cancer cachexia. Myostatin family ligand inhibition can prevent muscle atrophy in cancer cachexia, leading to a notable gain in muscle mass in comparison to cancer cachexia(Benny 61). MSTN may contribute to the process of muscular atrophy in cachexia cancer, which can result in a reduction in muscle mass. It’s possible that circulating MSTN does not significantly contribute to the development of cachexia cancer. Both normal and cachexic animals showed enhanced muscle development when myostatin expression was inhibited *in vivo* using RNA oligonucleotides in studies of muscle atrophy brought on by MSTN. The antisense approach is a viable treatment option for muscular dystrophy, and RNA oligonucleotides may suppress myostatin via the MyoD pathway. In HeLa cells of cervical cancer, MSTN knockout using CRISPR/Cas9 technology can induce apoptosis, whose mechanism is linked to changes in mitochondrial function, increased production of reactive oxygen species (ROS), release of cytochrome c, and activation of caspase (62). MSTN knockdown or knockout can inhibit the proliferation of tumor cells, such as liver cancer, cervical cancer, and other cells. Following MSTN knockdown, cell proliferation significantly decreased. Furthermore, apoptosis can be partially reversed by the addition of antioxidants or fatty acid oxidation inhibitors, indicating that the ROS-mediated mitochondrial pathway is crucial to this process. Many inhibitors, including peptidosomes and antibodies, are now being researched and developed to target MSTN. Lean body mass increased and fat mass decreased in a clinical trial of patients with prostate cancer following therapy with the anti-MSTN antibody AMG 745. This suggests that MSTN inhibitors may be used in the clinical treatment of disorders associated with muscle atrophy. Although the effects and modes of action of various MSTN inhibitors varied, some of them can decrease muscle atrophy, enhance body composition, and boost muscle strength in animal models. Given that MSTN plays a dual role in muscles and tumor cells, it is anticipated that MSTN inhibitors will be used in conjunction with other anti-cancer treatments in the future to prevent tumor growth and treat issues like cancer cachexia-induced muscle consumption. However, the optimal combination therapy plan still requires investigation.

#### 1.3.5 Mechanism of action of TNF-α on cancer cells

Tumor necrosis factor-α (TNF-α) has dual effects on cancer cells, with the main effect being promoting tumor progression (63). Its action is achieved through mechanisms such as specific receptor binding, activation of downstream signaling pathways, and regulation of key molecules. It also has potential for clinical translation and targeted therapy. Firstly, TNF-α drives malignant progression in most malignant tumors (64). In prostate cancer, it upregulates phosphorylated -IKK-α/β and phosphorylated -p38 levels, alters the ratio of cytoplasmic p50/IKK-α, increases p50 and p65 content, and activates the NF-κB pathway to promote tumor cell survival and malignant progression. In breast cancer, it enhances the invasion and metastasis ability of ER/PR positive cancer cells, and its effect is closely related to the activation of NF-κB and MAPK/ERK pathways, accelerating proliferation, migration, invasion and angiogenesis. In colorectal cancer, ovarian cancer, liver cancer, lung cancer, melanoma, glioblastoma, head and neck squamous cell carcinoma, pancreatic cancer, it regulates cancer cell proliferation, migration and invasion, and participates in tumor resistance and angiogenesis; it promotes the progression of chronic lymphocytic leukemia, affects tumor occurrence and bone marrow hematopoiesis; in multiple myeloma, it is associated with high cell division rate, low apoptosis rate and migration ability (65, 66).

Secondly, it only has tumor inhibitory effects under specific conditions. High-dose exogenous TNF-α can destroy tumor blood vessels and indirectly induce cancer cell necrosis; in some breast cancer cell lines (such as MCF-7), it can inhibit cell proliferation; in some prostate cancer cells, it can directly induce cell apoptosis. The core molecular mechanism of TNF-α acting on cancer cells is receptor-mediated downstream signal pathway activation. TNF-α activates downstream signals by binding to TNFR1 and TNFR2. After binding to TNFR1, it recruits adaptor proteins to form complex I, activating NF-κB, JNK, p38 MAPK and other pathways; the NF-κB pathway is the core of promoting tumors, which can upregulate the expression of inflammatory-related genes and anti-apoptotic genes; the MAPK pathway (ERK, JNK, p38) regulates cell proliferation, apoptosis and migration: ERK activation can inhibit the activity of apoptotic proteins, and p38 activation has cell-specific regulation of apoptosis and cell cycle. The regulation of epithelial-mesenchymal transition (EMT) through NF-κB upregulates transcription factors such as Snail, inducing the EMT process, significantly enhancing the migration and invasion ability of breast cancer, colorectal cancer and other tumor cells. The regulation of cytokines and tumor microenvironment to induce the expression of factors such as vascular endothelial growth factor (VEGF), interleukin-8 (IL-8) and others promotes tumor angiogenesis and reshapes the tumor microenvironment. The regulation of microRNAs (miRNAs) to regulate miR-145, miR-19a and other miRNAs expressions, thereby affecting the apoptosis, EMT and metastasis process of cancer cells. The application and potential targets of TNF-α in cancer treatment have extremely strong systemic toxicity and are limited for clinical use; isolated limb perfusion (ILP) combined with mechlorethamine and other chemotherapy drugs can locally destroy tumor blood vessels and increase drug concentration, used for the treatment of soft tissue sarcoma of the limb and locally metastatic melanoma; the improvement of nanotechnology: recombinant human TNF-α (rhTNF-α) combined with gold nanoparticles can reduce systemic toxicity and improve drug delivery efficiency, showing therapeutic potential in animal models of pancreatic cancer and thyroid cancer, still requires further research. Developing inhibitors/regulators against NF-κB, AKT, etc., is a new strategy for cancer treatment.

#### 1.3.6 Mechanism of action of CTGF on cancer cells

**Figure 2:**
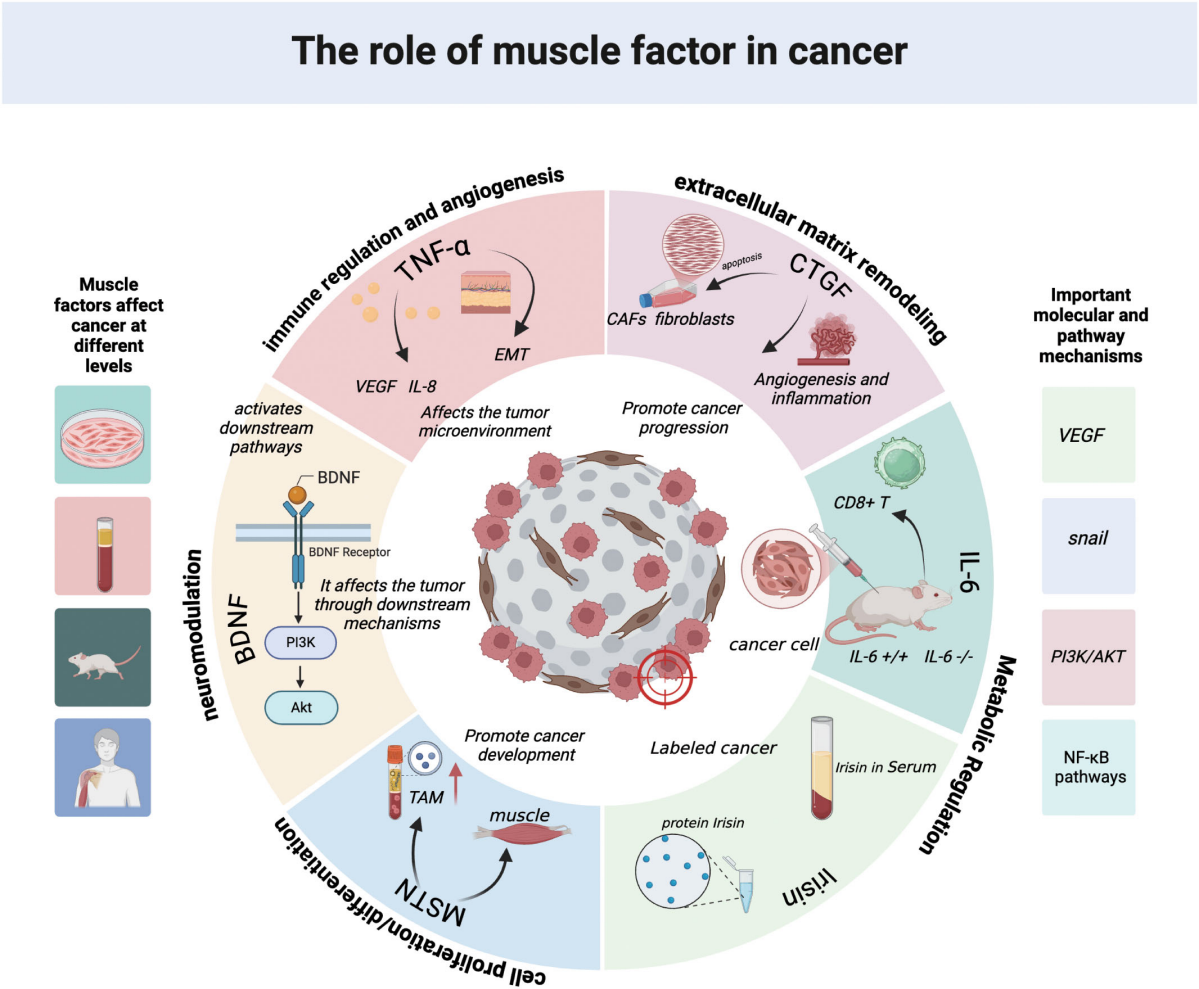
Multidimensional regulatory mechanisms of myokines in cancer myokines regulate cancer progression through multiple levels and pathways: at the immunoregulation and angiogenesis level, factors such as TNF-α, VEGF, and IL-8 affect the tumor microenvironment and induce epithelial-mesenchymal transition (EMT); at the extracellular matrix remodeling level, CTGF promotes angiogenesis and inflammatory responses by activating cancer-associated fibroblasts (CAFs); at the metabolic regulation level, IL-6 affects tumor progression by regulating the function of CD8^+^T cells; at the neuroregulation level, BDNF activates downstream signals through the PI3K/Akt pathway and affects tumors; at the cell proliferation and differentiation level, MSTN and irisin respectively regulate cell proliferation and tumor-related metabolism. These processes involve key molecules such as VEGF and snail, as well as core pathways such as PI3K/AKT and NF-κB. They play a crucial regulatory role in cancer occurrence and development from multiple dimensions including the tumor microenvironment, tumor cell behavior, and host metabolic immunity. The pictures in this article created in biorender (https://biorender.com/).

Connective tissue growth factor (CTGF/CCN2) is an extracellular matrix protein that regulates the interaction between cells and the extracellular matrix. In the tumor microenvironment, CTGF is a key driver of fibrosis and matrix hardening, which facilitates tumor invasion. CTGF binds to integrins (such as αvβ3), LRP (transmembrane proteins), and heparan sulfate proteoglycans (HSPG) (67). Through these interactions, it amplifies TGF-β signaling and promotes the transformation of fibroblasts into tumor-associated fibroblasts (CAFs). These CAFs secrete collagen and further secrete CTGF, forming a hard extracellular matrix, which enhances the migration of tumor cells through mechanotransduction pathways (such as YAP/TAZ) (67–69). CTGF also promotes angiogenesis by interacting with VEGF and inducing the migration and proliferation of endothelial cells. Systematic studies on CTGF have revealed strict environment-dependent characteristics based on cancer types. In breast cancer, pancreatic cancer, and gastric cancer, overexpression of CTGF is closely related to poor prognosis, metastasis, and disease progression (70–72). In these cancers, CTGF triggers epithelial-mesenchymal transition, angiogenesis, and drug resistance. In colorectal cancer and non-small cell lung cancer (NSCLC), some studies have shown that CTGF can act as a tumor suppressor. For example, in colorectal cancer, higher CTGF expression is associated with a higher overall survival rate in the data cohort figure 2 and table 1, which may be achieved by degrading β-catenin to inhibit metastasis (**Figure 2**; **Table 1**) (73, 74).

**Table 1:** Functional classification of muscle factors and their mechanism of action in cancer.

Myokine	Functional classification	Mechanisms in cancer
Irisin	Metabolic Regulation	Inhibits tumor cell proliferation, migration, and invasion via PI3K/AKT, EMT, and HIF-1α pathways.
IL-6	Metabolic/Immune Regulation	Promotes tumor growth and metastasis through STAT3, NF-κB, and MAPK pathways; regulates immune cells (Tregs, MDSCs) in the TME.
BDNF	Neuromodulation	Promotes tumor cell proliferation and invasion via TrkB-mediated PI3K/AKT and RAS/ERK activation.
MSTN	Cell Proliferation/Differentiation	Promotes muscle wasting and tumor progression; inhibits muscle growth via TGF-β pathway and recruits TAMs for immunosuppression.
TNF-α	Immune Regulation	Promotes tumor inflammation and angiogenesis via NF-κB and JNK pathways.
CTGF	ECM Remodeling	Enhances tumor cell migration, invasion, and angiogenesis via NF-κB and HIF-1α; modulates CAF metabolism.

### 1.4.The muscle factor irisin as a marker of cancer affects the prognosis of cancer

#### 1.4.1 Irisin as a potential biomarker for cancer

Irisin can be used as an auxiliary detection indicator for cancer prognosis, and the level of irisin in serum can also serve as an auxiliary diagnostic marker. There is a significant difference in irisin levels between bladder cancer patients and healthy individuals. Setting precise critical values can improve the sensitivity and specificity of irisin for bladder cancer diagnosis, and it is also helpful for tumor staging and grading (75, 76). It is widely expressed in breast cancer tissues, and the serum irisin level of breast cancer patients is much lower than that of the healthy control group. Studies have shown that a higher expression level is associated with a longer overall survival period for patients, and irisin is an auxiliary factor for predicting the survival of breast cancer patients (77). A study of 50 patients with head and neck cancer (88% male, 12% female) showed that the irisin level in patients receiving radiotherapy for head and neck cancer was lower. After evaluating the sample data, it was found that the irisin level in patients with renal cancer was significantly higher than that in the control group, indicating that irisin is also a potentially useful biomarker in renal cancer (78). Irisin is also significantly expressed in colorectal cancer and is related to tumor staging (79). In thyroid cancer, the synthesis level of irisin in eosinophilic mutant cells is significantly higher than in other thyroid tumors, assisting in the classification of thyroid tumors. At the same time, irisin is associated with high prognosis and low risk of malnutrition, and a higher irisin level may indicate malnutrition and be related to the onset and progression of cancer cachexia(de 80).

#### 1.4.2 IL-6 as a potential biomarker for cancer

IL-6 can be used as a potential biomarker for cancer diagnosis, prognosis assessment, and treatment monitoring (81). IL-6 plays a significant role in the occurrence, development, prognosis, and treatment of cancer, and is a key target in cancer research and treatment (82). Abnormal expression of IL-6 has been detected in various cancers, such as breast cancer (83), colorectal cancer (84), prostate cancer, ovarian cancer, lung cancer, pancreatic cancer, multiple myeloma, lymphoma, melanoma, cholangiocarcinoma, etc. Additionally, the levels of IL-6 in tumor tissues and serum of patients are usually significantly higher than those in normal tissues and healthy individuals. For example, in cholangiocarcinoma, the IL-6 level in the serum of patients is higher than that in hepatocellular carcinoma and metastatic colorectal cancer. In cholangiocarcinoma, the elevated IL-6 level in the preoperative serum is associated with a shorter disease-free survival period and poorer disease-specific survival rate. In intrahepatic cholangiocarcinoma with vascular invasion, the serum IL-6 level is significantly elevated, while it is significantly reduced after surgery or photodynamic therapy. In breast cancer, ovarian cancer, prostate cancer, lung cancer, pancreatic cancer, and other cancers, the IL-6 level is also closely related to disease progression, metastasis, and poor prognosis, such as in patients with metastatic breast cancer, the circulating IL-6 level can predict the survival status of the patients (85).

#### 1.4.3 BDNF as a potential biomarker for cancer

BDNF expression levels are correlated with patient outcomes in a number of cancer types. High expression of BDNF is typically linked to a lower patient survival and prognosis in lung cancer, breast cancer, liver cancer, colorectal cancer, malignant pleural mesothelioma (86), and other cancers. It may also be a useful biomarker of cancer prognosis. Positive expression was found in 71.8% of the samples for lung cancers such as squamous cell carcinoma and adenocarcinoma, and the high expression was substantially connected with the T-stage and histological type. In breast cancer, its expression is associated with adverse pathological and clinical outcomes. In liver cancer, it is intimately related to the angiogenesis and growth of tumors. It is closely linked to the growth and angiogenesis of tumors in liver cancer. Serum BDNF levels in patients with colorectal cancer were much lower than those in the control group, and these levels were linked to both patient prognosis and tumor metastasis (87). It is a distinct indicator of malignant pleural mesothelioma and is more prevalent in tumor tissue and pleural effusion than in benign samples or other malignancies. It has been demonstrated that overexpression of BDNF in human malignancies, particularly in paracancerous, lung, breast, and prostate tissues, is linked to a worse prognosis and increased mortality. Reduced BDNF expression was linked to better early survival but worse later outcomes in mouse models of Apc mutant intestinal/colon cancers. It also revealed a little drop in the number of small intestine polyps and an increase in colon polyp formation (88). BDNF was linked to PE-induced angiogenesis but had no effect on the carcinogenic potential of MPM cells. A new therapeutic target and MPM biomarker is BDNF. In LUAD, brain-derived neurotrophic factor is linked to BMS and is a potential prognostic indicator in and of itself. BDNF may be an immune-related biomarker and molecular target in LUAD patients, and it is intimately linked to TAM polarization of tumor-associated macrophages (89). Plasma BDNF may serve as a predictive.

biomarker of cancer-related cognitive impairment (90). As a result, BDNF measurement may benefit cancer patients, whether it is used to detect the disease or forecast how well a treatment would work.

#### 1.4.4 MSTN as a potential biomarker for cancer

Cancer cachexia patients’ plasma MSTN concentration was substantially lower than that of non-cachexia patients, and it was inversely connected with their loss of muscle mass and weight (91). But in some tumor tissues, like gastric, lung, and esophageal cancer tissues, the expression of MSTN protein is elevated. This is much higher than in para-cancer tissues, and it could be because of different post-transcriptional mechanisms or stages of the disease. In tumor circumstances, MSTN has a distinct function. A poor prognosis is predicted by elevated myostatin expression, which also causes muscle loss and accelerates tumor growth in lung cancer tissues. Patients with obesity or cancer may have circulating MSTN that reflects their muscle mass strength. Hypomuscular obesity (SO) or cancer cachexia (CC) are inversely correlated with MSTN. MSTN as a biomarker of muscle mass and strength in individuals with obesity or cancer, as well as a prostate cancer and cancer screening method (92).

#### 1.4.5 TNF -αas a potential biomarker for cancer

In breast cancer, the content of TNF-α in tumor tissues is significantly increased, and its expression is related to the clinicopathological features of tumor size, stage, lymph node metastasis (93). In liver cancer, TNF-α is highly expressed in cancer cells and immune cells in the tumor microenvironment, which is closely related to the malignancy degree and poor prognosis of the tumor. Serum TNF-α levels were significantly increased in some cancer patients, such as patients with invasive breast cancer, whose mean serum TNF-α was higher than that of the control group, and in patients with larger maximum tumor diameter, later TNM stage, and more advanced lymph node status (94–96). In clinical studies, TNF-α levels have been negatively correlated with the number of intratumor DCS, suggesting that it can be used as a potential prognostic marker for cancer patients. Prognosis assessment: The expression level of TNF-αis closely related to the prognosis of cancer patients. In clinical studies of multiple cancers, patients with high expression of TNF-α tend to have shorter survival, higher recurrence rates, and poorer treatment response. For example, in patients with colorectal cancer, high expression of TNF-α in tumor tissue is an independent adverse prognostic factor, which can be used to predict disease progression and survival of patients, providing an important reference for clinical treatment decisions (97).

#### 1.4.6 CTGF as a potential biomarker for cancer

CTGF (Connective Tissue Growth Factor) is aberrantly expressed in various malignancies, including colorectal cancer, melanoma, breast cancer, gastric cancer, pancreatic cancer, endometrial cancer, Hodgkin lymphoma, large B-cell lymphoma, and gallbladder cancer. The expression levels of CTGF in these cancers exhibit significant deviations from those observed in normal tissues and are closely associated with tumor stage, metastasis, and prognosis. Consequently, the expression level of CTGF may serve as a prognostic indicator. In the context of colorectal cancer, CTGF has been identified as a predictor of peritoneal metastasis, with lower expression levels correlating with a higher rate of peritoneal recurrence and reduced survival (98). In melanoma, elevated CTGF expression is linked to tumor progression, indicating a poor prognosis. Similarly, in breast cancer, CTGF is highly expressed in triple-negative breast cancer (TNBC) cell lines and tissues, with high levels correlating with unfavorable relapse-free survival outcomes (69, 73). In gastric cancer, CTGF expression in tumor tissues surpasses that in normal tissues and is positively correlated with tumor stage, peritoneal spread, lymph node metastasis, and poor prognosis. In pancreatic cancer, CTGF is predominantly expressed in cancer-associated fibroblasts (CAFs) and tumor cells, with its expression level correlating with the degree of tumor fibrosis. Furthermore, CTGF expression serves as a prognostic factor for tumor progression and survival in glioma patients. In endometrial cancer, CTGF overexpression is recognized as an independent prognostic factor, and it can also function as a biomarker to differentiate high-risk groups among patients with stage III-IV endometrial cancer. Elevated CTGF levels are known to promote the progression of endometrial cancer, positioning it as a novel prognostic biomarker for patient survival. While the CTGF gene may play a significant role in the early stages of colorectal cancer, it is not highly expressed in advanced cases, suggesting its potential utility as an early biomarker for risk stratification. In colorectal cancer, increased CTGF expression in tumor tissues is associated with overall survival and tumor-free survival among patients (99, 100).What’s more, dysregulation of CTGF is linked to enhanced cellular mechanisms that support tumor cell survival. This pattern of increased CTGF expression correlating with better prognosis is also observed in Hodgkin lymphoma, diffuse large B-cell lymphoma, and gallbladder cancer (101).

## 2 Conclusion

Through a complicated molecular network, muscle factor, a multifunctional signaling protein released by skeletal muscle, has a dual regulatory role in the development of cancer. Exercise encourages the release of muscle factors, which are derivatives also made under cancerous situations. The functions of muscle factors vary according on their source. In conclusion, IL-6 and TNF-α encourage tumor immune escape and the creation of an inflammatory milieu through the STAT3/NF-κB pathways, whereas irisin demonstrates broad-spectrum anticancer potential by blocking the PI3K/AKT, EMT, and HIF-1αpathways. While MSTN increases muscle consumption and attracts immunosuppressive cells via the TGF-β pathway, BDNF stimulates tumor cell invasion by activating the TrkB receptor. By controlling CAF metabolism and ECM remodeling, CTGF stimulates tumor angiogenesis and metastasis. MSTN inhibitors and anti-IL-6 therapies have advanced to the clinical trial stage, indicating the potential to improve cancer cachexia and boost the effectiveness of immunotherapy. Clinical evidence also indicates that muscle factors like Irisin, IL-6, and CTGF can be used as diagnostic and prognostic markers for a variety of cancers. Even though we now have a better understanding of the mechanism underlying the role of muscle factors, more research is still required to determine the precise regulatory network of muscle factors in various cancer types and disease stages. Additionally, customized treatment plans based on the expression profiles of muscle factors must be developed. For instance, using Irisin to increase chemotherapy sensitivity for precisely targeted therapy, or combining immune checkpoint inhibitors with anti-IL-6 antibodies for cancers with high IL-6 expression. The contradicting findings of muscle factors in various research (such the role of Irisin in specific malignancies) must be resolved at the same time, and its clinical significance must be confirmed using large samples and consistent experimental design. Create new biologics (such neutralizing antibodies or long-acting muscle factor analogues) and investigate how they work in conjunction with conventional treatments to hasten the transfer from lab to clinic. Develop individualized exercise intervention programs, elucidate the molecular mechanism of exercise-induced muscle factor secretion on cancer prevention and rehabilitation, and fortify the integration of sports medicine and oncology.
